# Respirometry reveals major lineage-based differences in the energetics of osmoregulation in aquatic invertebrates

**DOI:** 10.1242/jeb.246376

**Published:** 2023-10-31

**Authors:** Jamie K. Cochran, Catelyn Banks, David B. Buchwalter

**Affiliations:** ^1^ Department of Biological Sciences, North Carolina State University, Raleigh, NC 27695, USA; ^2^ North Carolina School of Science and Mathematics, 1219 Broad St, Durham, NC 27705, USA

**Keywords:** Aquatic invertebrates, Salinity, Metabolic rate, Aquatic insects, Calcium, Ion transport

## Abstract

All freshwater organisms are challenged to control their internal balance of water and ions in strongly hypotonic environments. We compared the influence of external salinity on the oxygen consumption rates (*Ṁ*_O_2__) of three species of freshwater insects, one snail and two crustaceans. Consistent with available literature, we found a clear decrease in *Ṁ*_O_2__ with increasing salinity in the snail *Elimia* sp. and crustaceans *Hyalella azteca* and *Gammarus pulex* (*r*_5_=−0.90, *P*=0.03). However, we show here for the first time that metabolic rate was unchanged by salinity in the aquatic insects, whereas ion transport rates were positively correlated with higher salinities. In contrast, when we examined the ionic influx rates in the freshwater snail and crustaceans, we found that Ca uptake rates were highest under the most dilute conditions, while Na uptake rates increased with salinity. In *G. pulex* exposed to a serially diluted ion matrix, Ca uptake rates were positively associated with *Ṁ*_O_2__ (*r*_5_=−0.93, *P*=0.02). This positive association between Ca uptake rate and *Ṁ*_O_2__ was also observed when conductivity was held constant but Ca concentration was manipulated (1.7–17.3 mg Ca l^−1^) (*r*_5_=0.94, *P*=0.05). This finding potentially implicates the cost of calcium uptake as a driver of increased metabolic rate under dilute conditions in organisms with calcified exoskeletons and suggests major phyletic differences in osmoregulatory physiology. Freshwater insects may be energetically challenged by higher salinities, while lower salinities may be more challenging for other freshwater taxa.

## Teaser

**Summary:** The influence of salinity on metabolic rates of freshwater invertebrates differs, perhaps as a result of evolutionary history.

## INTRODUCTION

Freshwater ecosystems support a disproportionally large amount of the Earth's biodiversity (Tickner et al., 2020; Williams–Subiza and Epele, 2021; Dudgeon et al., 2006). Within these systems, salinity ranges naturally from practically deionized to ion rich (Bates et al., 2018) as a function of geology, rainfall, evaporation and hyporheic influences (Cañedo-Argüelles et al., 2013; Iglesias, 2020). There are two dimensions of freshwater salinity change that appear to have significant ecological consequences. One dimension, which appears to be predominant, is the increasing salinization of freshwater, resulting from changing climate (Barlow and Reichard, 2010; Kinzelbach et al., 2003; Post, 2005; Werner et al., 2013; Dore, 2005) and human activities such as hydraulic fracking (Entrekin et al., 2011), mountain-top coal mining (Pond et al., 2008) and irrigation of arid landscapes (Allison et al., 1990; Halse et al., 2003; MacDonald et al., 2016). The second dimension involves systems becoming more dilute, as a result of, for instance, historic acid deposition (Driscoll et al., 2001; Jeffries et al., 2003; Johnson et al., 1969; Likens and Buso, 2012). It is apparent that both increasing and decreasing salinity regimes can have profound effects on biodiversity (Pond et al., 2008; Kefford, 2018; Minshall, 1969; Kaushal et al., 2018; Olson and Hawkins, 2017; Willoughby and Mappin, 1988), but we do not yet understand how the physiological attributes of different freshwater species, and lineages, determine how salinity change affects performance at the organismal level.

Regardless of whether freshwater aquatic organisms originated from terrestrial ancestors (e.g. insects) (Misof et al., 2014; Wootton, 1988; Wootton and Clark, 1972) or invaded freshwaters from marine habitats (e.g. crustaceans) (Vermeij and Dudley, 2000), they face the same physiological challenge of maintaining an internal environment (blood or hemolymph) that is strongly hypertonic to the surrounding medium. All freshwater animals must mitigate the inevitable effects of ion loss and water uptake (Silver and Donini, 2021; Bradley, 1987; Nowghani et al., 2017; Jonusaite et al., 2017; Kumai and Perry, 2012), with some combination of regulating cuticular permeability and taking up ions against concentration gradients at the expense of ATP consumption (Silver and Donini, 2021; Resh et al., 2008; Cochran and Buchwalter, 2022). To date, the energetic costs of osmoregulation in freshwater invertebrates remain poorly studied and major phyletic differences have yet to be revealed.

Aquatic insects typically thrive in relatively dilute conditions and often dominate fresh waters in terms of biomass, diversity and function (Covich et al., 1996; Huryn and Wallace, 2000; Merritt and Cummins, 1996). Very few species of aquatic insects can survive in saline waters (Bradley, 1987; Cochran et al., 2023; Herbst, 1999), and some entire lineages, such as mayflies, are being largely extirpated from anthropogenically salinized systems (Pond et al., 2008). Aquatic insects are considered secondarily aquatic and derived from terrestrial ancestors (Misof et al., 2014; Kristensen, 1981), and it appears that actively regulating their internal salinity is a common attribute, even when physiologically stressed by increases in salinity (Cochran et al., 2023; Dowse et al., 2017; Buchwalter et al., 2019; Scheibener et al., 2017). In contrast, studies suggest that some freshwater non-insect taxa, many of which have marine ancestry, may be challenged by the same dilute conditions that freshwater insects thrive in (Rukke, 2002; Hessen, 2000; Durant and Donini, 2018). Thus, despite facing the shared physiological challenges that life in freshwater imposes, there are apparently fundamental species differences in osmoregulatory processes that remain poorly understood.

The literature provides contrasting information about the relationships between ionic concentrations and uptake rates. Organisms may have ion influx rates that are proportional to diffusive ionic loss rates, which are greatest when the concentration gradient is largest between hemolymph/blood and the surrounding water. For example, studies in rainbow trout show decreased calcium uptake in higher salinity waters (Perry and Wood, 1985). However, freshwater insect ion uptake rates appear to uniformly increase with ion concentration (Cochran and Buchwalter, 2022; Cochran et al., 2023; Nguyen and Donini, 2010; Orr et al., 2021) (J. K. Cochran, S. E. Orr, D. H. Funk, W. P. Robarge, A. C. Figurskey, M. H. Reiskind and D. B. Buchwalter, unpublished), with ion turnover rates potentially affecting the energy budgets of aquatic insects in waters with highly elevated major ions. This apparent energetic cost might explain developmental delays, reduced growth and other fitness consequences associated with higher salinities (Buchwalter et al., 2019; Stobbart and Shaw, 1974; Williams and Feltmate, 1992; Verberk et al., 2020; Johnson et al., 2015).

Existing data generally indicate that higher metabolic rates are associated with lower salinities in most freshwater species, implying a greater energetic cost of offsetting diffusive ionic losses in progressively dilute situations. This metabolic response to salinity has been seen in snails, gammarids and other freshwater organisms with marine ancestry (McMahon and Russell-Hunter, 1978; Hudson, 1975; Duncan and Klekowski, 1967; Paolucci and Thuesen, 2020), but has not yet been linked to the transport of a specific ion. However, in aquatic insects, we could only find a single paper exploring the linkage between metabolic rate and salinity in the salt-tolerant mosquito *Aedes aegypti* (Edwards, 1982). Curiously, Edwards (1982) reports that oxygen consumption is unaffected by environmental salinity. Given that we have ample evidence for a universal positive association between ionic concentration and uptake rates of calcium, sodium and sulfate in aquatic insects (Cochran and Buchwalter, 2022; Cochran et al., 2023; Nguyen and Donini, 2010; Orr et al., 2021) (J. K. Cochran, S. E. Orr, D. H. Funk, W. P. Robarge, A. C. Figurskey, M. H. Reiskind and D. B. Buchwalter, unpublished), we might expect that aquatic insect metabolic rates might increase with rising salinity to meet the increasing energetic demands of ion transport (as seen in African clawed frog, caridean shrimp and other estuarine species (Schlieper, 1929; Peña-Villalobos et al., 2016; Wood et al., 2002; Allan et al., 2006; Ding et al., 2014).

Here, we asked whether fundamentally different physiological responses to salinity exist between aquatic insects and other aquatic species. We explored relationships between salinity, ion transport rates and oxygen consumption in several species (including three aquatic insect species and three other aquatic species – two crustaceans, *Hyalella azteca* and *Gammarus pulex*, and one snail, *Elimia* sp.). These species were specifically chosen to compare aquatic insects (with terrestrial ancestry) to species of non-insects (with marine ancestry) known to have a metabolic response to salinity. Specifically, we used respirometry to measure metabolic rate in each species in response to waters with different conductivities. Further, we assessed whether movement of specific ions may be more energetically costly, by performing ion flux experiments using radiotracers in waters with different conductivities and ion compositions.

## MATERIALS AND METHODS

### Animal rearing and collection

*Neocloeon triangulifer* (McDunnough 1931) (mayfly) were reared using previously described methods (Cochran and Buchwalter, 2022; Orr and Buchwalter, 2020). Briefly, *N. triangulifer* (WCC-2 clone) was originally isolated for culture from White Clay Creek (WCC), Chester County, PA, USA, and rearing methods were developed at the Stroud Water Research Center (SWRC; Avondale, PA, USA) (Sweeney and Vannote, 1984). At North Carolina State University, cultures were maintained in 4-qt (∼3.8 l) glass Pyrex dishes, lined with WCC periphyton plates) (Sweeney and Vannote, 1984) and aerated to maintain oxygen saturation. Naiads were reared at 21–23°C, on a 14 h:10 h light:dark photoperiod from egg hatch until they were relatively mature (approximately 3 weeks old) and approximately equal in size.

*Hydropsyche betteni* Ross 1938 (caddisfly) was collected from a riffle flowing out of Yates Mill Pond in Raleigh, NC, USA (35.718°, −78.686°) by removing larvae from rock bottoms with forceps. Animals were transferred back to North Carolina State University in a mason jar with mesh substrate and acclimated in ASW at room temperature.

*Drunella cornutella* (McDunnough 1931) (mayfly) were collected from the Basin Creek in Hays, NC, USA (36.375°, −81.144°) using a D-frame kick net in riffles, and animals were transferred back to NC State University in an aerated cooler. On arrival, they were acclimated in ASW at room temperature.

*Hyalella azteca* (Saussure 1858) (scud) were purchased from Goliad Farms (Goliad, TX, USA), packaged with ice packs, and shipped overnight to North Carolina State University.

*Gammarus pulex* (Linnaeus 1758) were ordered from Critters Direct (New Bern, NC, USA). On arrival, all organisms were acclimated in artificial soft water (ASW) at room temperature with dried leaves for food.

*Elimia* sp. (snail) were collected from Beaver Creek in Greensboro, NC, USA (35.999°, −79.712°) by removing specimens from rocks with forceps. Live snails were packaged with ice packs and transported to North Carolina State University for experimental use. Snails were acclimated in ASW at room temperature.

**Fig. 1.**
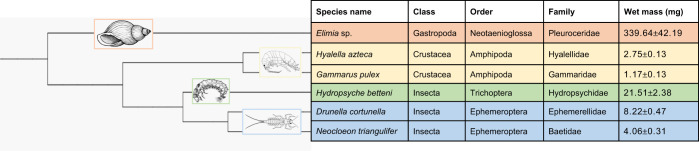
**Dendrogram showing the taxonomic structure of the species used in these experiments.** The dendrogram was generated using phyloT software; class, order, family and wet mass are shown for each species.

A dendrogram (Fig. 1) was assembled using phyloT (https://phylot.biobyte.de/), which automatically generated the tree based on the NCBI taxonomy database and visualized in iTOL (https://itol.embl.de/). Branch lengths are arbitrary and only represent taxonomic structure.

### Water preparation

CrystalSea Marine Mix (MM; Marine Enterprises International, Baltimore, MD, USA) was added to reverse-osmosis deionized water to create the desired experimental concentration (see Table 1). Waters were monitored for 24 h to ensure the desired final conductivity.

**Table 1.**
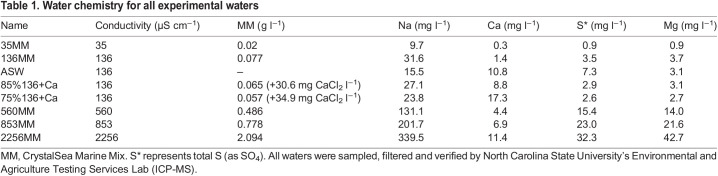
Water chemistry for all experimental waters

For waters where conductivity was held constant and Ca content was manipulated, we diluted the 136MM water with DI water (either 85% or 75% 136MM by volume) and titrated CaCl_2_ until the conductivity of the water reached 136 µs cm^−1^ and remained constant for 24 h.

ASW is our routine culture media and was prepared as previously described (Cochran and Buchwalter, 2022; Edwards, 1982).

### Respirometry

We used an 8-channel, fiber-optic, intermittent flow respirometry system (Loligo Systems, Tjele, Denmark) with AutoResp (v.2.1.0; Loligo Systems) software to obtain standard metabolic rate (SMR) measurements. Chambers (1.3±0.1 ml) were fitted with a glass spacer ring and stainless-steel mesh, which kept organisms separated from the continuously stirring magnet glass stir bar. Each chamber and corresponding fiber-optic channel recorded measurements of oxygen consumption for one individual. Chambers were submerged in an aerated, temperature-controlled water bath (∼4 l). Water bath temperature was controlled using a programmable heater/chiller (12108-30; Cole Palmer, Vernon Hills, IL, USA), which cycled water between the respirometry bath and a heat exchange coil submerged in the heater/chiller reservoir for the entire experiment to maintain a temperature of 20°C. For each experimental run (in which 8 individuals were evaluated), background oxygen consumption measurements (*n*=3–4 15 min cycles) were first taken from empty respirometry chambers using the intermittent respirometry setting in AutoResp 2.0. A Gilson Minipuls peristaltic pump (Gilson Inc., Middleton, WI, USA) refreshed the chamber medium with surrounding bath water (ASW) at the initiation of each 15 min cycle (300 s flush, 350 s wait, 250 s measure). Then, an individual was introduced into each of 8 chambers and intermittent flow respirometry was performed for 2–3 cycles (30–45 min). Individuals underwent no prior acclimatization or fasting.

After 2–3 cycles, the AutoResp software was changed to the ‘closed’ setting and chambers were no longer refreshed by the peristaltic pump until the oxygen level in the chambers dipped to around 80% O_2_. Previous experiments have suggested that oxygen saturation above 80% O_2_ appears to not be stressful in most species as this is well above most species’ critical oxygen tension (*P*_crit_) at 20°C (Cochran et al., 2022). When the oxygen levels in the chambers dipped to around 80% O_2_, the peristaltic pump was turned back on to refresh the chambers back to 100% O_2_. After at least 1 h, the source water for 4 of the 8 chambers was changed to a different treatment water (see Table 1). The same protocol was repeated with half of the chambers maintained in ASW and half exposed to the treatment water (oxygen level dipping to 80%, then being refreshed to 100%) for at least 2 h. This was repeated twice for each treatment (yielding *n*=16 for ASW for each species and *n*=8 for other treatments).

AutoResp software reports *Ṁ*_O_2__ in µg g^−1^ h^−1^, and accounts for differences in O_2_ capacity of water due to salinity (when different salinity is specified in the software). A one-way ANOVA and Tukey's multiple comparisons test found no significant differences between runs for a species, so all runs were combined for future analyses. We used the (q0.2) approach (Reemeyer and Rees, 2019), which uses quantiles to place SMR above the lowest 20% of observations for each individual before kPa fell below 10.5 (which corresponded with the relatively flat portion of each run for each individual).

To analyze effects of salinity, data were plotted using GraphPad Prism (v.6, GraphPad Software, La Jolla, CA, USA). Differences among salinities were assessed using a one-way ANOVA and Tukey's multiple comparisons test. All salinities were compared with every other salinity. Values are presented as means±s.e.m. unless otherwise indicated.

### Ion flux experiments

Individuals were 19 days old for all ^22^Na and ^45^Ca uptake experiments. Radioactive experimental waters were made with each treatment water, spiked with ^45^CaCl_2_ or ^22^NaCl (PerkinElmer, Billerica, MA, USA). Exposure activities ranged from 130 to 220 Bq ml^−1^. Exposures were measured with a PerkinElmer Wallac Wizard 1480 Automatic Gamma Counter (Shelton, CT, USA) or Beckman LS6500 Multipurpose Scintillation Counter (Beckman Coulter, Brea, CA, USA) immediately before the experiments began.

Beakers (100 ml high-density polyethylene) with 15 ml of radioactive exposure water were gently aerated, sealed with ParaFilm™ and spatially randomized. Each beaker held three individuals, one per time point (3, 6 and 9 h for dual-labeled with ^22^NaCl and 12, 24 and 36 h for ^45^CaCl_2_) and there were eight replicate beakers. At each time point, individuals were removed from the radioactive exposure waters by gently pipetting them into a mesh strainer (collecting any residual radioactive water in a waste container) and gently blotting dry. The organisms were then rinsed in two consecutive water baths of the corresponding unlabeled exposure water to remove loosely adsorbed ions from the exoskeleton. For ^45^Ca experiments, individuals were also rinsed with 0.05 mol l^−1^ EDTA and 0.1 mol l^−1^ l-ascorbic acid sodium salt because of the adsorptive nature of Ca on exoskeletons (Poteat and Buchwalter, 2014). Individuals were then blotted dry, weighed and digested in 500 μl of Soluene 350 (PerkinElmer) in a 20 ml glass vial at 28°C. After 48 h, they were neutralized with 500 μl of glacial acetic acid and 12 ml of scintillation cocktail (PerkinElmer Ultima Gold uLLT).

The uptake of each ion was plotted against time and uptake rates were calculated as the slopes of linear regression analysis using GraphPad Prism. Mass-specific calculations were based on wet mass. We applied appropriate corrections for spill-over and quench, and only measurements with lumex values <5% and error rates <10% were used in analyses. Flux rates were compared among groups using a one-way ANOVA with Tukey's multiple comparisons test using GraphPad Prism.

## RESULTS

### Respirometry

**Fig. 2.**
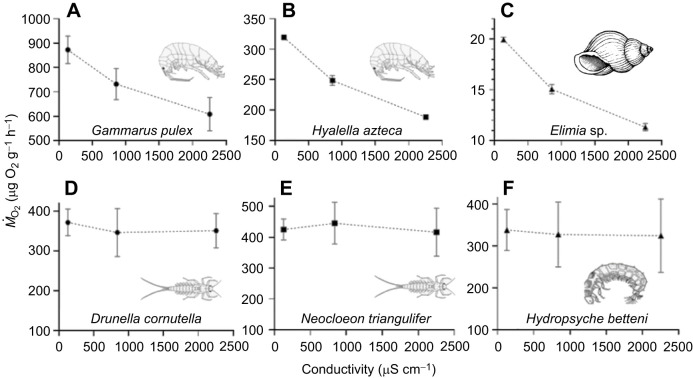
**The influence of salinity on oxygen consumption rate (*Ṁ*_O_2__) of aquatic invertebrates.** Lineage-specific patterns emerge, with crustaceans (A,B) and a snail (C) showing increased oxygen consumption with decreasing salinity, and mayflies (D,E) and a caddisfly (F) showing no change [*n*=16 for artificial soft water (ASW); *n*=8 for other treatments].

There was an apparent decrease in *Ṁ*_O_2__ with increasing salinity in *G. pulex* (Fig. 2A), *H. azteca* (Fig. 2B) and *Elimia* sp. (Fig. 2C). For example, *G. pulex* had a strong negative correlation between water conductivity and *Ṁ*_O_2__ (*r*_5_=−0.97, *P*=0.14); however, the relationship was not statistically significant. Metabolic rate was unaffected by changes in salinity in *D. cornutella* (Fig. 2D), *N. triangulifer* (Fig. 2E) and *H. betteni* (Fig. 2F).

### Ion flux experiments

**Fig. 3.**
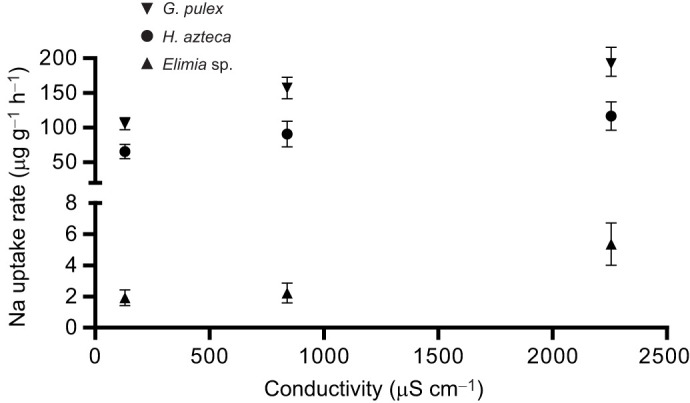
**Effect of salinity on sodium uptake rates.** Sodium uptake rates were positively associated with salinity and sodium concentration in *Gammarus pulex*, *Hyalella azteca* and *Elimia* sp. when exposed to a serial dilution. Data are means±s.e.m. (*n*=8).

There was an apparent increase in sodium uptake rates with increased salinity for *G. pulex, H. azteca* and *Elimia* sp., (Fig. 3). We found a strong positive relationship between sodium uptake rates and conductivity for *G. pulex* (*r*_3_=0.96, *P*=0.18), *H. azteca* (*r*_3_=0.98, *P*=0.11) and *Elimia* sp., (*r*_3_=0.96, *P*=0.16); however, the relationship was not statistically significant.

**Fig. 4.**
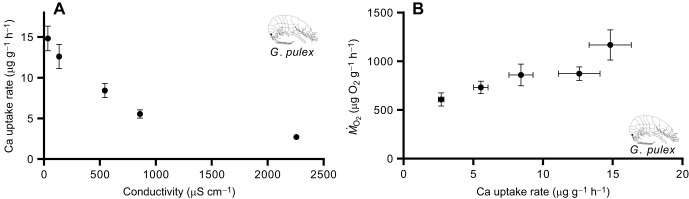
**Effect of salinity and *Ṁ*_O_2__ on calcium uptake rates.** (A) Calcium uptake rates were negatively associated with salinity and calcium concentration in *G. pulex* when exposed to a serial dilution. (B) Calcium uptake rates were positively associated with oxygen consumption rates in *G. pulex*. The same pattern was observed in *H. azteca.* Data are means±s.e.m. (*n*=8).

In MM waters, calcium uptake rates decreased with increased salinity for *G. pulex* (Fig. 4A), *H. azteca* and *Elimia* sp. (Table S1). *Ṁ*_O_2__ was also positively correlated with calcium uptake in *G. pulex* (*r*_5_=−0.93, *P*=0.02) (Fig. 4B), *H. azteca* (*r*_3_=−0.99, *P*=0.03) and *Elimia* sp. (*r*_3_=0.91, *P*=0.26) (Table S1).

**Fig. 5.**
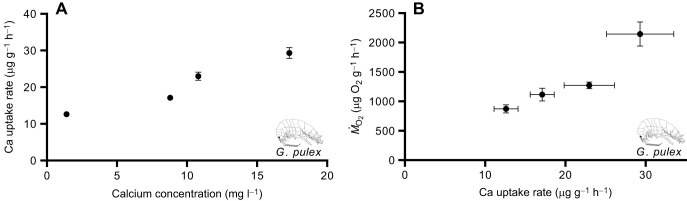
**Effect of calcium concentration and *Ṁ*_O_2__ on calcium uptake rates.** Calcium uptake rates were positively associated with (A) calcium concentration and (B) *Ṁ*_O_2__ in *G. pulex* when conductivity was held constant but calcium was varied from 1.7 to 17.3 mg l^−1^. Data are means±s.e.m. (*n*=8).

In *G. pulex*, when water conductivity was held constant, but calcium content was manipulated, calcium uptake rates were positively correlated with Ca concentration (*r*_4_=0.97, *P*=0.02) (Fig. 5A). Further, calcium uptake was positively correlated with metabolic rate (*r*_4_=0.94, *P*=0.05) (Fig. 5B).

## DISCUSSION

**Fig. 6.**
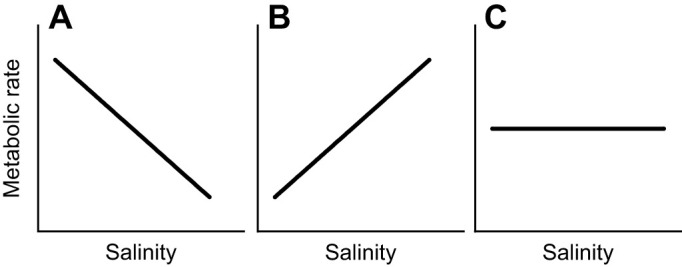
**Three models of energetics and fluxes in aquatic organisms.** (A) Metabolic rate is higher in dilute conditions and is associated with the increased need to offset diffusive losses (e.g. snails). (B) Metabolic rate increases with salinity to meet the increasing energetic demands of ion transport (e.g. observed primarily in some marine/euryhaline species). (C) Metabolic rate does not change with salinity (e.g. aquatic insects).

Existing literature indicates lower salinities are associated with increased metabolic rates in gammarids, snails, common carp and cutthroat trout (McMahon and Russell-Hunter, 1978; Hudson, 1975; Duncan and Klekowski, 1967; Paolucci and Thuesen, 2020; Shokri et al., 2019; Wolvekamp and Waterman, 1960; Morgan and Iwama, 1999; De Boeck et al., 2000) (Fig. 6A). These findings suggest a greater energetic cost of offsetting diffusive ionic losses in progressively dilute situations in these species. However, ecological (Pond et al., 2008; Kefford, 2018) and organismal/physiological (Scheibener et al., 2017; Poteat and Buchwalter, 2014; Scheibener et al., 2016; Buchwalter et al., 2019) studies indicate more saline conditions are more stressful to most aquatic insects, even when given the opportunity to acclimate (Cochran et al., 2023; Orr and Buchwalter, 2020). Thus, despite the common need for all freshwater organisms to regulate their internal environments in hypotonic surroundings, there are clearly physiological differences among lineages that are yet to be described.

Therefore, we first explored relationships between salinity and oxygen consumption rates in six species – three insects (from terrestrial ancestry) and three non-insects (from marine ancestry) –across a gradient of salinity. Commensurate with previous studies (McMahon and Russell-Hunter, 1978; Hudson, 1975; Duncan and Klekowski, 1967; Paolucci and Thuesen, 2020), we found that *G. pulex*, *H. azteca* and *Elimia* sp. had decreased metabolic rate with increasing salinity. Though not studied here, previous work has shown some estuarine and brackish water species demonstrate increased metabolic rate with increasing salinity (potentially to meet the increasing energetic demands of ion transport) (Schlieper, 1929; Peña-Villalobos et al., 2016; Wood et al., 2002; Allan et al., 2006; Ding et al., 2014; Aguilar et al., 1998) (Fig. 6B). However, this is not the case for all euryhaline species; for example, there is evidence for decreased oxygen consumption with increased salinity in the euryhaline tilapia (*Oreochromis mossambicus*) (Sparks et al., 2003). Here, we show for the first time that aquatic insects do not show a metabolic response to salinity. We therefore propose a third potential scenario for the energetic impact of osmoregulation in hypotonic environments (Fig. 6C). In this scenario, organisms may have increased ion fluxes with increasing salinity; however, unlike the scenario in Fig. 6B, metabolic rate may not change with salinity. Here, it appears that aquatic insects primarily rely on shifting their energetic stores to meet the increased demands of ion transport, rather than altering their metabolic rate to cope with increased salinity, leading to delays in growth and development (Johnson et al., 2015; Buchwalter et al., 2019). To our knowledge, a similar metabolic response to salinity has only been seen anecdotally in aquatic insects, in the salinity-tolerant mosquito *Aedes aegypti* (Edwards, 1982), the Chinese mitten crab (Schwabe, 1933), the European eel (Raffy, 1993), brine shrimp (Gilchrist, 1956) and the giant freshwater prawn (Ern et al., 2013).

Next, we asked whether the changes in metabolic rate observed with increased water conductivity in the three non-insect species could be linked to the transport rates of a particular ion. We found that Na uptake rates were positively correlated with Na concentration and associated conductivity (Fig. 3). This is consistent with previous work in aquatic insects, where a positive association between Na uptake and the Na concentration of the water is the rule (J. K. Cochran, S. E. Orr, D. H. Funk, W. P. Robarge, A. C. Figurskey, M. H. Reiskind and D. B. Buchwalter, unpublished) (Cochran and Buchwalter, 2022; Cochran et al., 2023; Orr et al., 2021; Scheibener et al., 2016). However, Na uptake rates (Fig. 3) were negatively correlated with metabolic rate (Fig. 2), which suggests that Na uptake does not explain the increased energetic cost for these species under dilute conditions. In contrast, we found that Ca uptake was negatively associated with conductivity for all three non-insect species in these experimental waters. This finding differs strongly from all previous insect studies we have done, where we have always observed a positive association between Ca uptake and Ca concentration (J. K. Cochran, S. E. Orr, D. H. Funk, W. P. Robarge, A. C. Figurskey, M. H. Reiskind and D. B. Buchwalter, unpublished). In *Daphnia*, acclimation to dilute water has been shown to increase Ca uptake (Durant et al., 2018). However, our findings are consistent with other studies in rainbow trout (*Salmo gairdneri*) and tilapia (*Oreochromis mossambicus*), where increased salinity was associated with decreased calcium uptake (Wood et al., 2002; Ding et al., 2014).

One interpretation of our data is that under dilute challenge, non-insect species (or those with calciferous exoskeletons) prioritize the increased uptake of Ca despite its apparent energetic cost (e.g. increased Ca uptake is stimulated by dilute challenge). An alternative, though not mutually exclusive possibility is that other ions (e.g. Na, SO_4_) have a suppressive impact on Ca uptake rates when those ionic concentrations increase. An example of this type of suppression was observed by Scheibener et al. (2017), who showed that Na is antagonistic to SO_4_ transport in *N. triangulifer*.

We provide two lines of evidence that link calcium uptake rate to metabolic rate in non-insects. First, we demonstrate a stimulation of calcium transport under increasingly diluted conditions that was linked to increased metabolic rates. This may be associated with energetic costs of increasing calcium transport to offset diffusive losses. Second, when we held total conductivity constant (136 µS cm^−1^) and manipulated calcium concentration from 1.7 to 17.3 mg l^−1^, we saw a positive association between calcium uptake rate and metabolic rate. This finding suggests that calcium transport in these animals is largely linked to ATP consumption. However, we did not observe this pattern in aquatic insects. Why are they so different?

One possibility is that the rates of ion transport do not change in response to salinity. We reject this notion, based on several studies which show that aquatic insect transport rates appear to always be concentration dependent. For example, Scheibener et al. (2016) found that sodium transport was positively associated with sodium concentration in *H. betteni*. Scheibener et al. (2017) found that sulfate uptake was positively associated with sulfate concentration for *N. triangulifer*, *Drunella* sp., *Ephemerella* sp., *Isonychia* sp. and *Maccaffertium* sp. (Scheibener et al., 2017). A recent study has found a positive association between calcium, sodium and sulfate uptake and calcium, sodium and sulfate concentration (respectively) in eight species of aquatic insect species (including mayflies, caddisflies and mosquitoes) (J. K. Cochran, S. E. Orr, D. H. Funk, W. P. Robarge, A. C. Figurskey, M. H. Reiskind and D. B. Buchwalter, unpublished). While these studies show that this is the case in naive animals (e.g. animals exposed to short-term or abrupt salinity changes), we have also shown that animals will acclimate to high salinity conditions by reducing uptake rates towards control rates, though their uptake rates are still concentration dependent. For example, calcium, sodium and sulfate uptake rates are positively associated with sulfate ion concentrations (respectively) in *N. triangulifer* after chronic exposure to elevated ion treatments (Orr et al., 2021). Similar trends have also been seen in *Callibaetis floridanus* after chronic exposure to a wide range of salinity treatments (Cochran et al., 2023). We also saw an acclimatory change in *N. triangulifer* exposed to progressively more dilute matrices, with increases in transport rates observed in acclimated animals that more closely approximate those of animals in standard rearing conditions than those of animals at higher salinity (Cochran and Buchwalter, 2022).

A second possibility is that aquatic insects maintain somewhat constitutive expression of ion-transporting ATPases and utilize secondary transporters to modulate overall uptake rates when salinity changes. However, proteomic data from the gills of *N. triangulifer* exposed chronically to different salinities show that V-ATPase, Na^+^/K^+^-ATPase and Ca^2+^-ATPase expression changes (Orr et al., 2023). *Neocloeon triangulifer* and *Callibaetis coloradensis* show increases in ionocytes (mitochondria-rich osmoregulatory structures) with more dilute rearing condition (Cochran and Buchwalter, 2022; Wichard et al., 1973). *Aedes aegypti* also show increases in mitochondria in the anal papillae with dilute rearing conditions (Edwards, 1982). More studies are needed to further address the function and response of these transporters.

A third possibility is that aquatic insects shift the allocation of their energetic resources to osmoregulation at the expense of growth and development (Johnson et al., 2015; Buchwalter et al., 2019). We are still confronted with the conundrum of the energetic linkages between osmoregulation and metabolism. One perspective is that the most energetically favorable situation for aquatic organisms is to have blood that is isotonic to the external media (Potts, 1954). This is clearly not the case in insects, which appear to thrive in dilute environments and suffer when the external environment approaches isotonicity with the internal media (Kefford, 2018; Dowse et al., 2017). It is tempting to speculate that the terrestrial derived aquatic insect osmoregulation system is optimized to very large transmembrane potentials between apical cells and the external media but to our knowledge this has never been measured.

Intriguingly, we generally see very low rates of Ca uptake in aquatic insects relative to Na and SO_4_ and often must use longer exposure durations in our Ca uptake studies. It appears that relatively slow Ca uptake is a common feature of aquatic insects, which have a chitinous exoskeleton (as opposed to calciferous in crustaceans) (Hessen, 2000; Orr and Buchwalter, 2020). In a comparative Ca study radiotracer with Hydropsychidae and Ephemerellidae, Poteat and Buchwalter (2014) found Ca uptake rates were so slow that it was necessary to lower stable Ca exposure concentrations to facilitate uptake of ^45^Ca. Further, Orr et al. (2021) provided histological evidence for possible Ca damage to Malpighian tubules under Ca-rich conditions, suggesting that excessive Ca uptake is potentially toxic in aquatic insects. In contrast, Ca is a significant component of crustacean exoskeletons and molluscan shells (Zanotto and Wheatly, 2002; Jeziorski and Yan, 2006; Zehmer et al., 2002; Briers, 2003), and those species appear to increase their metabolic rates to support Ca uptake under dilute conditions.

It remains unclear to what extent these phyletic differences are linked to the evolutionary origins; however, we posit that the evolutionary history of these species may have some impact on their physiological response to salinity. Freshwater systems are composed of organisms from many different lineages. Phylogenomic analysis of aquatic insects shows that there is no clear common ancestor, but rather that multiple invasions of water from land occurred over millions of years (Misof et al., 2014; Wootton, 1988; Wootton and Clark, 1972). In contrast, 30 clades of crustaceans, such as the scuds *G. pulex* and *H. azteca*, have invaded freshwater from marine origins (Vermeij and Dudley, 2000). Likewise, all living freshwater mollusks also evolved from marine origins (including 15 gastropod clades) (Vermeij and Dudley, 2000).

Here, we posit that there are three distinctive relationships among salinity and metabolic rate in aquatic organisms (Fig. 6). In the first, organisms (such as gammarids) show decreased metabolic rate with increasing salinity (McMahon and Russell-Hunter, 1978; Hudson, 1975; Duncan and Klekowski, 1967; Paolucci and Thuesen, 2020). In the second, metabolic rate increases with increasing salinity (as seen in estuarine species) (Schlieper, 1929; Peña-Villalobos et al., 2016; Wood et al., 2002; Allan et al., 2006; Ding et al., 2014). In the third, we show for the first time that aquatic insects have no metabolic response to increased salinity. We also provide evidence that in non-insect species with a metabolic response to salinity, it appears as though Ca is the driver rather than Na. Altogether, we suggest that both the decreased demand for Ca and lack of metabolic response to salinity in aquatic insects could provide a physiological explanation for aquatic insects thriving in dilute environments, relative to other taxa. However, future work in non-insect aquatic lineages lacking a marine ancestry and/or a calciferous exoskeleton (or shell) would be informative in determining whether that relationship is a function of the biology of aquatic insects or ancestry.

## Supplementary Material

10.1242/jexbio.246376_sup1 Supplementary information
