# Supplementary material for: Respirometry reveals major lineage-based differences in the energetics of osmoregulation in aquatic invertebrates
Source: J Exp Biol. 2023 Oct 31;226(20):jeb246376. doi: 10.1242/jeb.246376 (PMC10629685; doi:10.1242/jeb.246376)
Supplement: Supplementary information [file jexbio-226-246376-s1.pdf]

Table S1. Calcium uptake rates for *H. azteca* and Elimia sp. when exposed to a serial dilution.

		H. azteca			**Elimia sp.**		
Name	Conductivity	mean	SEM	n	mean	SEM	n
136MM	136	25.07	0.88	8	1.60	0.81	8
ASW	136	41.71	2.80	8	2.48	0.69	8
853MM	853	20.45	2.21	7	0.34	0.17	8
2256MM	2256	8.92	3.20	8	0.30	0.64	8

Journal of Experimental Biology • Supplementary information
